# Evaluation of the decontamination efficacy of a portable air cleaner using 275-nm UVC-LED radiation against airborne Coronavirus and Influenza virus

**DOI:** 10.3205/dgkh000573

**Published:** 2025-08-15

**Authors:** Janina Reissner, Benjamin Reichelt, Paul Siller, Gerrid Brockmann, Martin Kriegel, Uwe Roesler, Anika Friese

**Affiliations:** 1 Institute of Animal Hygiene and Environmental Health, Veterinary Centre for Resistance Research – TZR, School of Veterinary Medicine, Freie Universität Berlin, Berlin, Germany; 2 Chemical and Veterinary Investigation Office Freiburg, Freiburg, Germany; 3 Animal Health and Animal Welfare, Faculty of Agricultural and Environmental Sciences, University of Rostock, Rostock, Germany; 4 Federal Office of Consumer Protection and Food Safety, Department Veterinary Drugs, Berlin, Germany; 5 Hermann-Rietschel-Institut, Technische Universität Berlin, Berlin, Germany

**Keywords:** air decontamination, airborne virus, COVID-19, influenza A virus, indoor air quality, portable air cleaners, UV radiation

## Abstract

**Background:**

The COVID-19 pandemic has highlighted the risk of airborne transmission of viruses, especially in public indoor spaces or healthcare settings. Effective indoor air purification systems are necessary to limit the spread of these pathogens, and the deployment of portable air cleaners (PACs) has increased rapidly since then. Germicidal ultraviolet (UV) radiation technologies have recently supplemented conventional air filtration technologies. Thus, the purpose of this study was to evaluate the air decontamination efficacy of a PAC using a 275-nm UVC-LED unit and fibrous-media air filters.

**Method:**

Two different filters were used in the study: a High-Efficiency Particulate Air (HEPA) filter and an Efficient Particulate Air (EPA) filter. The PAC was operated in an experimental aerosol chamber with an airflow rate of 200 m³/hour for 10 or 20 minutes. Subsequently, the concentration of infectious viruses and particles in the air was measured. Decontamination efficacy was compared between UVC-LED radiation, filtration, and a combination thereof against Feline Coronavirus (FCoV) and influenza A virus (H3N2) aerosols.

**Results:**

Infectious virus reductions were comparable between the UVC and filter measurements. A decrease of 94% in FCoV concentration was observed after 10 minutes of device runtime, increasing to 99.8% after 20 minutes compared to control measurements. H3N2 showed greater susceptibility, with a reduction of 99.7% achieved after 10 minutes. Interestingly, a synergistic effect was observed with significantly lower virus concentrations when both technologies were combined.

**Conclusion:**

These findings highlight the potential of PACs equipped with emerging UVC-LED technologies as effective tools for indoor air decontamination. The deployment of PACs equipped with UVC radiation and filtration could be a promising alternative or supplement to ventilation systems, especially in healthcare settings and other public spaces.

## Zusammenfassung

**Hintergrund:**

Die COVID-19 Pandemie hat das Risiko einer Übertragung von Viren über die Luft, insbesondere in öffentlichen Räumen und Gesundheitseinrichtungen, verdeutlicht. Effektive Raumluftreinigungssysteme sind notwendig, um die Ausbreitung dieser Krankheitserreger einzuschränken, und der Einsatz mobiler Luftreiniger hat seitdem stark zugenommen. Pathogen inaktivierende ultraviolette (UV) Strahlungstechnologien haben in letzter Zeit die konventionellen Luftfiltertechnologien ergänzt. Daher wurde in dieser Studie die Luftdekontaminationswirkung eines mobilen Luftreinigers, ausgestattet mit einer 275 nm UVC-LED Einheit und Luftfiltern, untersucht.

**Methode:**

In der Studie wurden zwei verschiedene Filtertypen eingesetzt: ein Schwebstofffilter (HEPA-Filter) und ein Hochleistungs-Partikelfilter (EPA-Filter). Der mobile Luftreiniger wurde in einer experimentellen Aerosolkammer mit einer Luftstromrate von 200 m³/h für 10 oder 20 min betrieben. Anschließend wurde die Konzentration infektiöser Viren und von Partikeln in der Luft gemessen. Die Dekontaminationswirkung wurde zwischen UVC-LED-Strahlung, Filtration und einer Kombination beider Technologien, für Aerosole des Felinen Coronavirus (FCoV) und Influenza-A-Virus (H3N2), verglichen.

**Ergebnisse:**

Die Reduktion infektiöser Viren war zwischen den UVC-Strahlung- und Filtermessungen vergleichbar. Nach 10 Minuten Betriebszeit des Geräts wurde eine Reduktion der FCoV-Konzentration um 94% beobachtet, die nach 20 Minuten Laufzeit auf 99,8% anstieg, verglichen mit Kontrollmessungen. H3N2 zeigte eine höhere Empfindlichkeit mit einer Reduktion von 99,7% bereits nach 10 Minuten. Interessanterweise wurde ein synergistischer Effekt mit signifikant niedrigeren Virenkonzentrationen bei gleichzeitiger Anwendung beider Technologien beobachtet.

**Schlussfolgerung:**

Die Ergebnisse unterstreichen das Potenzial von mobilen Luftreinigern, ausgestattet mit neuartigen UVC-LED Technologien, zur Dekontamination von Innenraumluft. Ihr Einsatz könnte eine vielversprechende Alternative oder Ergänzung zu Belüftungssystemen, insbesondere in Gesundheitseinrichtungen und anderen öffentlichen Räumen, sein.

## Introduction

The recent SARS-CoV-2 pandemic has underscored the global concern regarding the transmission of respiratory diseases through aerosols, particularly in crowded indoor environments. These circumstances have led to the development and evaluation of novel purification technologies as an alternative to conservative air filtration aimed at inactivating these viruses [1]. In 2021, the World Health Organization (WHO) officially acknowledged short- and long-range airborne transmission of SARS-CoV-2 through the inhalation of infectious aerosols, in addition to droplet and fomite transmission [2]. Previous field studies on aerosols have detected viable SARS-CoV-2 and SARS-CoV-2 RNA in hospitals and healthcare settings with COVID-19 patients [3], [4], [5], [6]. Moreover, animal trials with hamsters and ferrets have demonstrated airborne transmission of SARS-CoV-2 [7], [8], [9]. Beyond SARS-CoV-2, numerous other respiratory viruses, such as influenza A virus, Middle East respiratory syndrome Coronavirus (MERS-CoV), respiratory syncytial virus (RSV), or human rhinovirus (hRV) are known to be transmitted through aerosols [10], [11], [12], [13], [14]. Infectious aerosol particles vary in size and quantity and are generated during respiratory activities, such as the coughing or even breathing of an infected person [15], [16], [17]. Notably, most of these particles are smaller than 5 µm, allowing them to remain suspended in the air for hours and access the lower respiratory tract [18], [19], [20].

The stability of pathogens in the environment is influenced by factors such as temperature, relative humidity, UV light, and oxidative stress. These factors play a significant role in determining the potential for these pathogens to be transmitted [21], [22]. Previous studies have shown that SARS-CoV-2 and Influenza A virus remain infectious in aerosols for several hours [23], [24], [25]. Respiratory viruses also remain infectious on surfaces for several hours to days and can be re-aerosolized [26]. These findings highlight the increased risk of airborne transmission in poorly ventilated indoor areas, where superspreading may occur [4], [27], [28].

While recommended precautions such as physical distancing, mask-wearing, hand hygiene, and surface disinfection partially mitigate aerosol transmission, the need for effective indoor air purification systems is evident. Portable air cleaners (PACs) have emerged as a cost-efficient solution, especially in buildings lacking suitable heating, ventilating, and air-conditioning (HVAC) systems. PACs commonly equipped with fibrous-media air filters have shown promising results in reducing airborne pathogens, depending on where they were set up in rooms [29], [30]. In addition, ultraviolet (UV) radiation-based disinfection, particularly in the UVC spectrum (200–280 nm), has gained attention as a chemical-free and sustainable method for efficiently reducing pathogens on several surfaces and in the air, as shown in previous studies [31], [32], [33]. 250–270 nm is considered the optimum wavelength for microbial inactivation, as nucleic acids in pathogens can effectively absorb it [34]. The absorption of a photon leads to the formation of pyrimidine dimers between pyrimidine bases in the DNA/RNA strands, which leads to disrupted transcription and replication [32]. UVC light-emitting diodes (UVC-LEDs) emitting between 255 and 280 nm are an emerging technology in the medical field as an alternative to the most common but controversial mercury-vapor lamps, which emit at 254 nm [35], [36]. The development and marketing of many UVC-based disinfection products, like PACs with integrated UVC units, has increased significantly since the COVID-19 pandemic. However, in most cases, independent, standardized testing procedures and verification of their decontamination capability are lacking [37], [38]. This study assesses the air decontamination efficacy of a prototype PAC equipped with a 275-nm UVC-LED radiation unit and mechanical filter to address this lack. The effectiveness of this system was tested in a controlled chamber using aerosolized Feline Coronavirus (FCoV) and an influenza A virus strain (H3N2). 

## Materials and methods

### Virus and cell lines

FCoV, biotype FECV (isolate “München”, FLI, Insel Riems, Germany; viral registration number RVB-1259), was propagated using Crandell-Rees Feline Kidney Cells (CRFK; ATTC CCL-94) in Dulbecco’s Modified Eagle Medium (DMEM High Glucose, Biowest, Nuaillé, France) supplemented with 10% fetal bovine serum (FBS; PAN Biotech, Aidenbach, Germany) and 1% of a solution containing 100 IU/mL penicillin G, 100 µg/mL streptomycin (Biochrom AG, Berlin, Germany) and 25 µg/mL amphotericin B (Biozym, Hessisch Oldendorf, Germany), as described recently [39]. 

Influenza A virus (H3N2) strain A/Hong Kong/8/68 (ATCC-VR-1679) was propagated in Madin-Darby Canine Kidney cells (MDCK; ATCC-CCL-34). The cells were maintained in T-175 cell culture flasks (Sarstedt AG & Co. KG, Nürmbrecht, Germany) in Eagle’s minimal essential medium (EMEM, ATCC, Manassas, VA, USA) supplemented with 1.5% Panexin CD (Pan Biotech, Aidenbach, Germany), 0.5% FBS, and 1% penicillin-streptomycin-amphotericin B mix. Once the cells reached 100% confluence, the medium was removed, and the cells were incubated in EMEM without supplements. After 1 hour, this medium was removed, and 11 ml of virus propagation medium was added. The virus propagation medium consisted of EMEM with 5% Panexin CD and one µg/ml TPCK-trypsin (Life Technologies GmbH, Darmstadt, Germany). The cells were infected with 500 µl of H3N2 at a 10^6^ TCID_50_/ml concentration and incubated at 37°C in a 5% CO_2_ atmosphere. Cytopathic effect (CPE) was observed one day after infection, and cell culture flasks were frozen at –80°C. After one freeze-thaw cycle and a centrifugation step to remove cells, the supernatant concentration was determined and stored at –80°C. 

### Virus quantification

The virus concentrations of the original virus suspensions and air samples were determined using an endpoint dilution assay. Cells were grown to confluency in 96-well plates overnight. Afterward, cells were inoculated with 100 µl of 10-fold serial dilutions of air samples or original virus suspension. The medium for MDCK cells was changed to a virus propagation medium before inoculation. Each well was investigated for CPE five days after infection. A tissue-culture infectious dose 50 (TCID_50_/ml) was then calculated using the Spearman-Kaerber method [40], [41]. Before titration, air samples were filtered with filters of 0.22 µm pore size (Roth, Karlsruhe, Germany).

### Description of the portable air cleaner (PAC)

**Figure 1:**
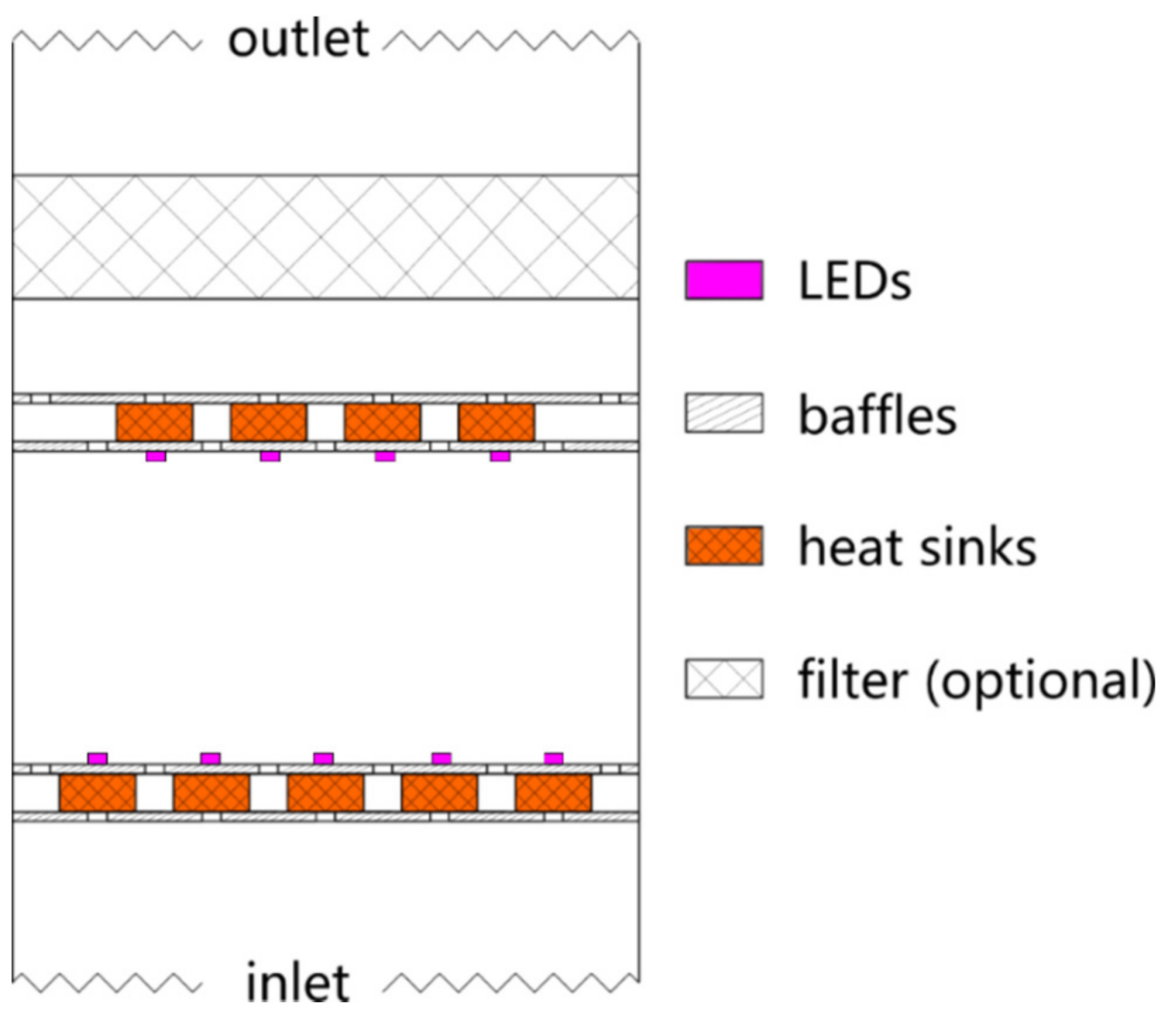
Components and their arrangement in the modified PAC

A PAC (OurAir TK 850 designed by MANN+HUMMEL GmbH, Ludwigsburg, Germany) was converted for the tests in the aerosol chamber. The original filter was removed and replaced with a UVC chamber (ams-OSRAM AG, Premstaetten, Austria), with the option of installing a smaller mechanical filter (see Figure 1 (Fig. 1)). The airflow enters by a fan in the lower part of the device and is forced through the UVC chamber and, if required, the filter. On entering the UVC chamber, the airflow is guided through two slotted baffle plates with an offset. The UVC chamber is 0.13 m long and has a 0.46 m x 0.46 m cross-section. The airflow in the chamber is homogeneous due to the evenly distributed arrangement of the inlet and outlet slots. The air is guided through two identically offset slotted baffle plates when it leaves the chamber. The air can be additionally filtered before flowing back into the aerosol chamber through an outlet grille. Two different mechanical fibrous-media air filters were used in the study according to the DIN EN 1822 standard: a High-Efficiency Particulate Air (HEPA) H14 filter and an Efficient Particulate Air (EPA) E11 filter. The filters have a depth of 90 mm.

In the UVC chamber, five rows of 21 LEDs (ams-OSRAM AG) are installed on the chamber inlet face and four rows of 21 LEDs on the outlet face. Each LED emits a radiant power of 63mW at a wavelength of 275 nm. The radiation can be switched on and off. The UVC chamber walls are coated with PTFE films, achieving a reflectivity of more than 80%. Heat sinks are installed between the baffles to dissipate the generated heat from the LEDs utilizing forced convection. The minimal volume flow rate is 200 m^3^/hour to ensure sufficient cooling. At this flow rate, a particle moves through the chamber at an average speed of 0.26 m/s and is thus irradiated for approx. 0.5 s. No radiation can escape the chamber. Therefore, influences due to changes in reflection boundary conditions can be excluded by using different or no filters.

### Experimental setup in the aerosol chamber

The PAC was installed in an airtight walk-in aerosol chamber with a volume of 7 m³. Further technical details, including an illustration of the aerosol chamber, were previously published [42]. In brief, original virus suspensions of FCoV or H3N2 were diluted to a concentration of 10^6.3^ TCID_50_/ml in DMEM with 10% FBS and transported to an ultrasonic nebulizer (Broadband Ultrasonic Generator, SonoTek Corporation, Milton, MA, USA) using a perfusor pump at a flow rate of 36 mL/hour. The ultrasonic nebulizer generated a bioaerosol with particles of an average initial size of 18 µm. After 10 minutes of nebulization, initial concentrations of infectious virus were measured, which served as a quality control of the virus-generating process. The particle concentration/m³ and particle size distribution were measured by an aerosol spectrometer (Grimm, model 1.109, GRIMM Aerosol Technik Ainring GmbH & Co., KG, Germany) placed in the middle of the chamber. We differentiated between the effect of novel 275-nm UVC-LED radiation and fibrous-media air filters (H14 and E11 filters) as a conventional technology for air decontamination. Additionally, the decontamination efficacy of a combination of the two technologies was investigated. 

To assess the performance of each technology, we first determined the virus concentration in the aerosol during device operation without UVC radiation or mechanical filtration. These control measurements represent the natural loss of the virus in the air, influenced by the ventilation of the PAC and the respective time. We studied the decontamination efficacy in two different variants in the experimental setup (V1 and V2). V1 represented a scenario where a virus shedder had already left the room. FCoV or H3N2 was aerosolized for 10 minutes to generate a concentrated viral aerosol in the chamber. Subsequently, aerosolization was stopped, and the device with the tested technology (UVC-LED or filter) was operated for 10 or 20 minutes, followed by an air sampling period. The device has a minimum airflow rate of 200 m³/hour. Theoretically, the chamber volume was passed through five times in 10 minutes and ten times in 20 minutes. The V2 setup served as a stress test, representing a scenario where a virus shedder is currently in the room. FCoV or H3N2 aerosolization took place for 10 minutes while the device with the respective technology was concurrently applied. After virus nebulization and decontamination ended, an air sample was collected. All experiments were conducted in quintuplicate. The E11 filter was only used for V1 and 10 minutes running time of the PAC to check for a difference between the filter types. Air samples were collected by a Coriolis µ cyclone air sampler (Bertin Instruments, Montigny-le-Bretonneux, France). The air sample volume was 3,000 L, using an airflow rate of 300 L/min over a sampling period of 10 min. The Coriolis µ cones were filled with 15 mL supplemented cell culture medium. For FCoV, DMEM with 1% of FBS was used; for H3N2, EMEM with 5% Panexin was used. In addition, 0.3% autoclaved linseed oil was added to prevent foam formation. As described previously, the air samples were stored on ice until quantification using an endpoint dilution assay.

### Statistical analysis

All experimental data were analyzed using SPSS software version 29.0 for Windows (SPSS, Inc., Chicago, IL). A one-way ANOVA was conducted to compare the efficacy of the different PAC technologies in each of the experimental settings (V1 10 min, V1 20 min, V2) in reducing the concentration of infectious virus in the air. The one-way ANOVA revealed that the FCoV and H3N2 concentrations differed statistically significantly (*p*<0.001) for the different decontamination technologies in each experimental setup. To determine whether and how technologies differed from each other, we carried out a contrast analysis in which we compared the virus concentrations (lg TCID_50_/m³) of the control measurements with all decontamination measurements, those of the individual technologies with those of the combined technologies, those of the UVC measurements with those of the filter measurements and finally the filter measurements with each other. *p*<0.05 was considered statistically significant. GraphPad Prism 8 (GraphPad Software, San Diego, CA, USA) was used to create all graphs. 

## Results

### Decontamination efficacy of the different PAC technologies on bioaerosols

A comparative analysis was performed to evaluate the decontamination efficacy on viral aerosols of a prototype PAC modified with a 275-nm UVC-LED chamber placed in front of the filter unit. The efficacy of UVC radiation, the efficacy of mechanical filters, as well as a combination of the two were examined in two scenarios (V1 and V2 setup), as described above. The average RH in the chamber was 33%, and the temperature measured 23°C throughout all experiments. 

**Figure 2:**
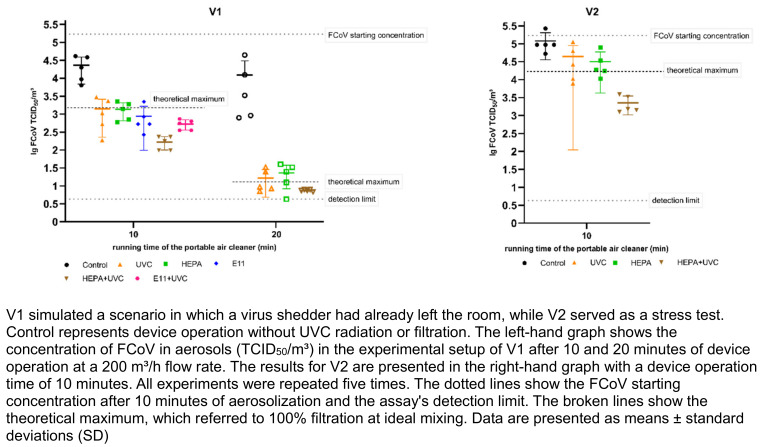
Decontamination efficacy of the different air decontamination technologies of the PAC (UVC-LED, HEPA filter, E11 filter and combinations) in relation to the concentration of infectious FCoV in aerosols tested for scenarios V1 and V2.

**Figure 3:**
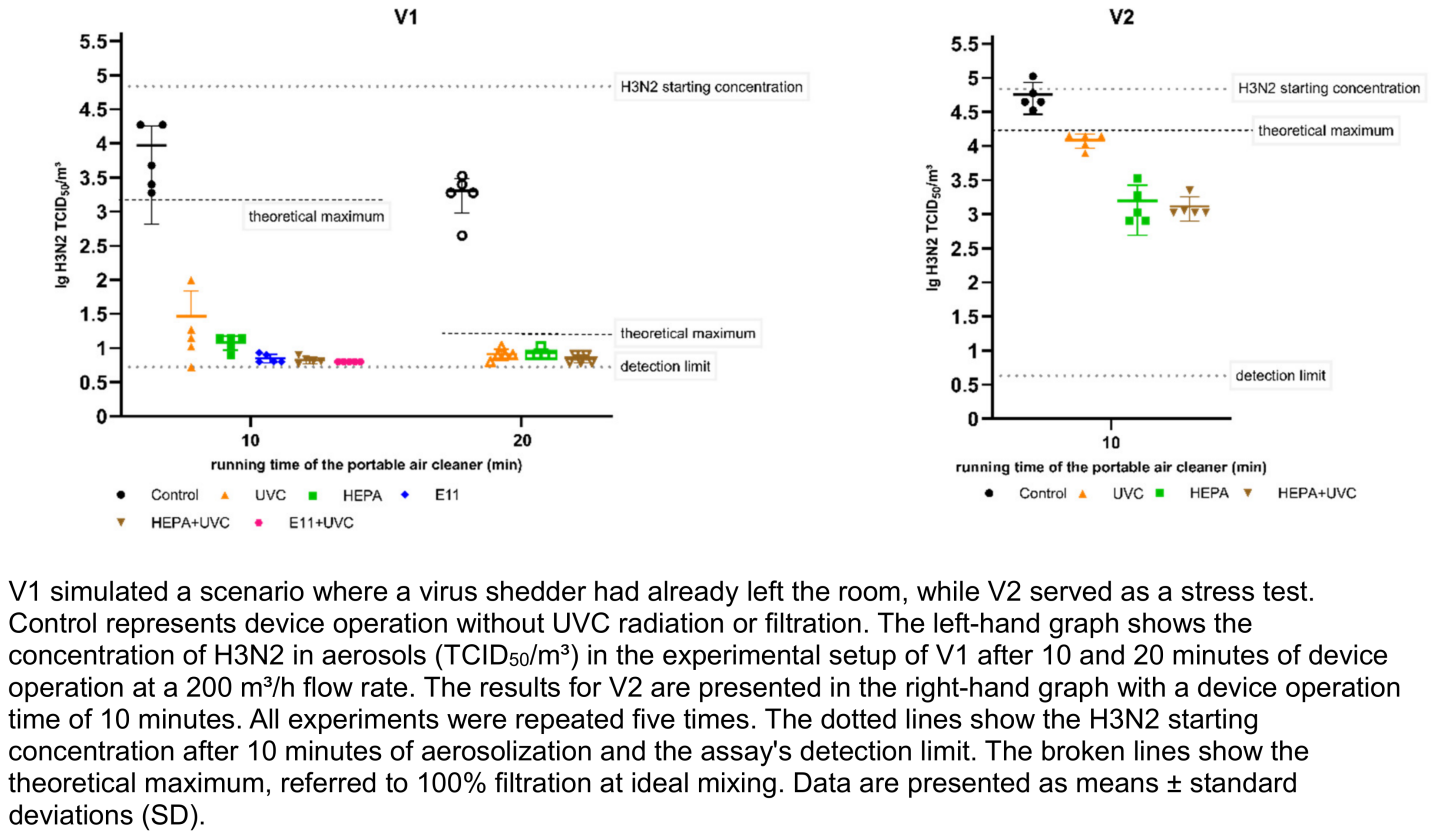
Figure 3. Decontamination efficacy of different air decontamination technologies of the PAC (UVC-LED, HEPA filter, E11 filter and combinations) in relation to the concentration of infectious H3N2 in aerosols tested for scenarios V1 and V2.

Figure 2 (Fig. 2) shows the decontamination efficacy of the PAC for FCoV aerosols using different decontamination technologies. The results for H3N2 aerosols in the same configurations are depicted in Figure 3 (Fig. 3). After 10 minutes of aerosolization, the average initial concentration of FCoV was 5.23±0.17 lg TCID_50_/m³ air. For H3N2, the average initial concentration was 4.83±0.22 lg TCID50/m³ air. Recovery rates of aerosolized FCoV and H3N2 were calculated by dividing the initial concentration of collected viruses per m^3^ of air by the theoretical virus concentration per m^3^ of air. The recovery rate was 13% for FCoV and 4% for H3N2. Analytical values of reduction with 100% filtration at ideal mixing, referred to as the theoretical maximum, were calculated from the initial concentration over time. In the V1 setup, the concentration would theoretically be reduced by 2.06 or 4.13 lg levels after 10 or 20 minutes of device operation, respectively. In the V2 setup it would be reduced by 1.01 lg. 

Control measurements were performed to quantify the concentration of infectious virus in the aerosol during device operation without the decontamination technologies. The initial concentrations decreased by 0.9 lg levels after 10 minutes for both viruses and by 1.1 (FCoV) or 1.5 (H3N2) lg levels after 20 minutes in the V1 setup. In the V2 setup, the control values were comparable to the initial concentrations, as the virus was added continuously during air treatment. Based on the control values, the lg reductions of the FCoV or H3N2 concentrations achieved by the PAC with the respective technology were calculated (see Table 1 (Tab. 1) and Table 2 (Tab. 2)). Contrast analysis was done in which the virus concentrations of the control measurements were compared with all decontamination measurements, those of the individual technologies with those of the combined technologies, those of the UVC measurements with those of the filter measurements, and the two filter measurements with each other. 

All decontamination measurements showed significantly lower virus concentrations for infectious FCoV and H3N2 than the control measurements in both setups (*p*<0.001). For FCoV aerosols, a reduction of over 99% was achieved after 20 minutes of PAC operation in all decontamination measurements in the V1 setup, while for H3N2 aerosols, this reduction was already achieved after 10 minutes of PAC operation. The lg reductions for H3N2 after 20 minutes were equivalent to those after 10 minutes. Since the control value was 0.67 lg lower after 20 minutes and the assay's detection limit was almost reached, in our system, we cannot show a higher lg reduction than 2.5 compared to the control. There was no statistically significant difference between the efficacy of UVC-LED and mechanical filtration for 10 or 20 minutes of PAC operation for FCoV (*p*=0.914; *p*=.416) and H3N2 (*p*=0.271; p=0.651) in the V1 setup. In the V2 setup, HEPA filtration and UVC radiation showed no efficacy differences for FCoV (*p*=0.821). For FCoV, no significant difference was found between the two filter types (*p*=0.227). However, for H3N2, we found a substantial difference between the two filter types (p=0.007). Interestingly, significantly lower virus concentrations were observed after 10 and 20 minutes when combining the technologies compared to using each technology individually for FCoV (*p*<0.001; *p*=0.043) and H3N2 (*p*=0.024; *p*=0.047) in the V1 setup. A positive effect was also observed in the V2 setup with the combination of UVC-LED and HEPA filtration for both FCoV and H3N2 (*p*<0.001) compared to the single technologies. For FCoV, the combination of the two technologies resulted in virus concentrations that were below the theoretical maximum reduction, whereas concentrations below the theoretical maximum were observed for H3N2 in all measurements.

### Particle concentration in the aerosol

**Figure 4:**
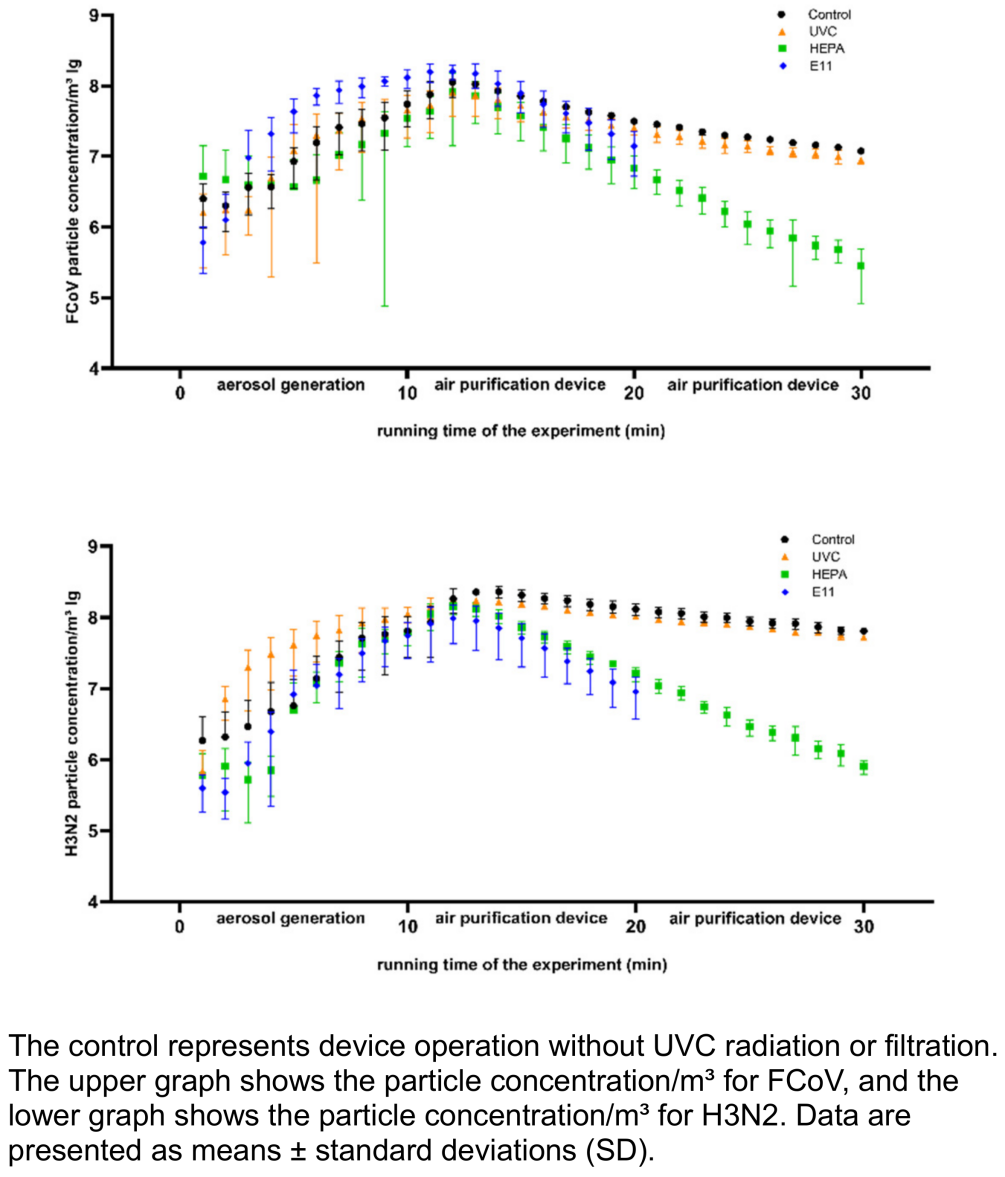
Particle concentration of FCoV and H3N2 per cubic meter air for PAC runtime (10 and 20 min) with UVC-LED, HEPA filter, and E11 filter

Mechanical filters decontaminate the air by removing virus-containing particles from it, while UVC technologies inactivate these particles. To verify this effect, we measured the particle concentration in the aerosol chamber using an aerosol spectrometer throughout all experimental trials. Figure 4 (Fig. 4) shows the development of the particle concentration per cubic meter of air for both FCoV and H3N2 during aerosol generation and running time of the PAC. FCoV aerosols reached their peak particle concentration after 12 minutes, measuring 8 lg particles/m³. In the control and with UVC-LED activation, the particle concentration dropped by 0.5 lg levels after 10 minutes and 1 lg level after 20 minutes. This corresponds to a reduction of 70% and 89%, respectively. In contrast, HEPA filtration and the combination of filtration with UVC-LED radiation reduced the particle concentration by 1 lg level after 10 minutes of operation and 2.5 lg levels after 20 minutes, which corresponds to reductions of 90% and >99%, respectively. The E11 filter exhibited trends similar to those of the HEPA filter. H3N2 aerosols also reached their peak particle concentration after 12 minutes, measuring 8.2 lg particles/m³. In the control and with UVC-LED activation, this concentration dropped by 0.3 lg levels after 10 minutes and 0.5 lg levels after 20 minutes. This corresponds to a 43% and 71% reduction of particles, respectively. HEPA filtration and the combination of filtration with UVC-LED radiation reduced the particle concentration by 0.9 lg levels after 10 minutes of operation and 2.3 lg levels after 20 minutes, corresponding to reductions of 88% and >99%, respectively. The E11 filter exhibited trends similar to those of the HEPA filter. Our aerosol spectrometer revealed that 97% of the particles for both FCoV and H3N2 aerosols were <5 µm, with the remaining 3% >5 µm. 

## Discussion

An independent evaluation of the efficacy of novel air purification technologies to combat airborne transmission of pathogens is an essential step toward improving public health. This study evaluated the air decontamination efficacy of a prototype PAC equipped with a 275-nm UVC-LED unit and a mechanical filter in a controlled setting. We compared the effectiveness of UVC-LED radiation and mechanical filtration as stand-alone methods and the combination of the two technologies in different experimental setups. Overall, we observed comparable efficacy between UVC radiation and mechanical filtration, with a notable synergistic effect when both technologies were combined.

We selected FCoV as a surrogate virus for SARS-CoV-2 for this study as it can be tested under BSL-2 conditions, as well as an H3N2 influenza A strain for the same reason. Corona- and influenza virus have been among the most critical and widespread respiratory pathogens that could spread over aerosols [10], [43], [44]. In a previous study, FCoV showed outstanding stability over hours in the airborne state, like SARS-CoV-2 [23], [45]. Influenza A viruses of human and avian origin also remained airborne and infectious for hours [46].

The study's two experimental settings, V1 and V2, allowed us to assess efficacy under varying viral load conditions, with V2 serving as a stress test featuring continuous virus generation. As infectious virus was still detectable in the air after 10 minutes of device operation in the V1 setup, we assessed its efficacy after 20 minutes, ensuring a minimum airflow rate of 200 m³/hour to prevent device overheating. The theoretical maximum reduction for ideal mixing ventilation with 100% filtration indicated a reduction of 99% or 99.99% of the initial virus concentration after 10 or 20 minutes of device operation in V1 setup. Notably, our control measurements in the V1 setup showed that the initial concentrations of our test viruses in the aerosol were already reduced by ≥90% after 10 minutes of PAC operation, solely from operating the PAC for 10 minutes without any specific decontamination measures. This reduction is likely due to the natural loss of infectivity over time, sedimentation of viral particles, potentially enhanced by air movement through the device and sampling methods [47], [48]. We hypothesize that viral aerosol particles impact in the device, adhere to chamber walls, or settle due to gravity. Additionally, the PAC affects the airflow in the chamber, which cannot be ideally mixed. All measurements for infectious viruses were similar to or significantly below the theoretical maximum, underscoring the loss of infectivity due to factors other than filtration. Therefore, we based our decontamination efficacy calculations on viral concentrations measured in the control rather than initial viral concentrations. This approach acknowledges effects that some studies and manufacturers may neglect. 

Nevertheless, in both experimental settings, all decontamination measurements exhibited significantly lower virus concentrations than did the controls. Considering the stand-alone decontamination efficacy of 275-nm UVC, we observed a reduction in the concentration of infectious FCoV by 94% after 10 min and by 99.8% after 20 minutes in the V1 setup. In the stress test, a reduction of 63% was achieved. Barnewell et al. [49] tested a 254-nm UVC filtration system and found no infectious SARS-CoV-2 in the airflow directly behind the system after 10 minutes of operation. Nicolo et al. [50] reported a reduction in SARS-CoV viral RNA copies of 95% after 60 minutes and 99% after 75 minutes using a 254-nm UVC device in a small box. Their study could not determine how long the virus was infectious. In our research for H3N2, a reduction of 99.7% was already achieved after 10 minutes, and in the stress test, it was reduced by 79%. McDevitt et al. [51] also observed high susceptibility of airborne influenza A virus (H1N1) to UVC light, consistent with our findings. Remarkably, we found no difference in infectious virus reduction between the UVC and filter measurements. Ueki et al. [52] tested the effect of HEPA filtration on airborne SARS-CoV-2, reporting reductions ranging from 85% to 99.9% with air changes of the chamber volume from 1 to 7.1 times. It is important to note that comparisons between these studies are challenging due to great variations in the experimental setups.

Mechanical filters decontaminate the air by removing virus-containing particles. Therefore, we additionally measured the particle concentration of the aerosol. We found 97% of the particles for both FCoV and H3N2 aerosols were smaller than 5 µm, with the remaining 3% being larger than 5 µm. H14 filters have to remove at least 99.97% of particles between 0.15 to 0.2 µm, while the E11 filter should remove 95% [53]. Interestingly, we found no difference between the two filter types. In the H14 and E11 filter measurements, we observed approximately 90% fewer particles/m³ air after 10 minutes and 99.5% fewer after 20 minutes. Fernstrom et al. [54] published similar results, that with 12 air changes per hour, it would take approx. 12 minutes to reduce airborne particles by 90% and 23 minutes to reduce them by 99% with HEPA filtration. Duill et al. [55] tested a PAC with HEPA filtration in a classroom and observed a reduced particle concentration of 90% in 30 minutes. It is essential to note that while the entire room volume circulates through the device within a specific timeframe, depending on the size of the room, the airflow pattern, and the airflow rate of the device, not every air molecule is necessarily affected. Analytically, the difference in the degree of separation of H14 and E11 filters with ideal mixing is logarithmically 2.06 to 1.96 for 10 minutes. HEPA filters have high initial and operating costs. They are extremely dense, which leads to high pressure losses. To compensate for the pressure losses, increased energy consumption is required. Since the electricity costs for device operation were identified as a potential barrier to its use, other filters should be considered [56]. Less dense filters, such as the E11 filter, might be an effective alternative in PACs, as our test setup revealed no significant differences in filtration performance and lower pressure losses might result in less energy consumption. 

The product performance guideline of the US Environmental Protection Agency (EPA) for air sanitizers [57] states that successful decontamination efficacy is only achieved when the virus concentration is reduced by 3 lg levels (99.9%). Although both air decontamination technologies demonstrated significantly lower virus concentrations compared to the control, this effectiveness was only reliably achieved when the two technologies were combined, namely after 10 minutes of device operation for H3N2 and after 20 minutes for FCoV. The minimum dose of virus particles that trigger an infection is assumed to be >10^3^ to 10^7^ TCID_50_ for different influenza A viruses and could also be assumed to be in this range for Coronaviruses [26], [58]. In this experimental setting, a 2 lg reduction (99%) of the initial virus concentration, which was obtained by all technologies after 10 minutes in V1, could already successfully minimize the infection risk. 

UV inactivation of pathogens depends on the UV radiation dose, calculated from the irradiance intensity of the source multiplied by the irradiation time. Extremely few studies dealing with UVC radiation dose exist, especially at 275-nm, for the inactivation of Coronaviruses or influenza viruses in aerosols. Due to different experimental setups, devices, and measurement techniques, it is not easy to compare the results [59], [60]. Viruses dried on surfaces are more resistant to UV radiation than viruses in aerosols, likely due to the protective formation of aggregates and biofilms on surfaces [61], [62]. Lee et al. [63] found that a 275-nm UVC-LED device achieved 99.99% SARS-CoV-2 elimination on surfaces with a UV dose exceeding 10 mJ/cm². For a 99.99% inactivation of FCoV on stainless steel carriers with 275-nm UVC-LED radiation, a dose of 80 mJ/cm2 was needed [64]. For inactivation of airborne SARS-CoV-2 at 254-nm, low doses ranging from 0.42 to 0.51 mJ/cm² have been suggested [61]. Abkar et al. [59] reviewed the literature up to 2022 on airborne virus inactivation with different UVC sources and found that a 90% reduction could be achieved with an average dose of 2 mJ/cm². The results of our study suggest that the radiation dose achieved is within this effective range. Extrinsic factors, e.g., ambient humidity, temperature, and the suspension medium, significantly influence viral inactivation rates. Higher humidity often decreases UV sensitivity due to increased water retention and retention of protective substrates such as salts or proteins [59], [65]. Our experiments were conducted under low humidity conditions at approximately 33%, as FCoV was found to be most stable in the aerosol at low humidity conditions previously [45]. The suspension medium consisted of cell culture medium supplemented with 10% FBS to mimic a high organic load similar to respiratory droplets, aiming to enhance airborne virus protection [66]. Intrinsic virus characteristics also affect UVC sensitivity. We observed that FCoV and H3N2 viruses showed different susceptibilities to UVC-LED radiation, with H3N2 being more susceptible, despite both being enveloped, single-stranded RNA (ssRNA) viruses. While ss viruses are generally more sensitive due to the absence of a second strand for damage repair, factors such as viral structure, molecular weight, and protein composition contribute to this difference [67]. The radiation dosages required for successful inactivation differ depending on the UVC wavelength and even among different Coronaviruses [64], [68]. Therefore, testing diverse pathogens is crucial for a comprehensive understanding of UVC inactivation rates. Interestingly, filtration also led to a greater reduction in infectious H3N2 compared to infectious FCoV. Additionally, for H3N2, all measurements were significantly below the theoretical maximum reduction. Since the particle measurements indicated no difference in separation between the H3N2 and FCoV aerosols, these observations suggest that H3N2 is inherently more sensitive in its aerosolized state. Supporting this hypothesis, recovery rates showed that three times more infectious FCoV than H3N2 were recovered in this aerosol setup. 

During the COVID-19 pandemic, PACs became popular in crowded, enclosed rooms as a quick, cost-effective emergency measure for air decontamination. Factors like room geometry, airflow patterns, optimal operational duration, device placement relative to the infection source, and the viral load require careful consideration to achieve sufficient decontamination performance with a PAC [38]. HEPA filters are the most studied air purification technology in air conditioning systems and devices [38]. PACs equipped with HEPA filters showed up to 80% efficacy in reducing SARS-CoV-2 RNA in air in field studies [69], [70]. Recently, PACs have been equipped with emerging UVC-based technologies in addition or as an alternative to conventional air filtration. However, the lack of independently standardized efficacy evaluation and verification of these devices remains an issue [37], [53]. Traditionally, mercury-vapor lamps at a germicidal wavelength of 254-nm were used for UVC radiation, but these pose health and environmental risks and have therefore been phased out [32], [63]. UVC-LEDs have emerged as safer and more efficient alternatives [35]. Studies investigating the efficacy of upper-room UVC-LED lamps at wavelengths around 275±10 nm have demonstrated promising results comparable to traditional mercury-vapor lamps for Coronavirus inactivation [63], [68], [71], [72]. It is essential to note that UVC light in the range of 250–280 nm can harm skin and eyes, depending on the irradiation intensity [32]. Far-UVC lamps at 222-nm and 233-nm have demonstrated efficacy against Coronavirus, while posing minimal risk to human health [64], [73]. However, it should be considered that their operation generates ozone, the quantity of which must be assessed with regard to a possible health risk, taking into account the specific application conditions [74]. Mitigating these risks requires careful utilization of UVC devices and lamps. In our test device, UVC-LEDs are securely installed within a chamber to prevent radiation from leaking. 

The aerosol experiments were conducted in a 7-m³ aerosol chamber approved for BSL-2 work. While the U.S. EPA’s product performance guideline for air sanitizers recommends a minimum chamber size of 23 m³ for air sanitizer testing, most studies use smaller environments (e.g., laminar flow cabinets, small boxes, or air ducts) due to the lack of standardized BSL test chambers, [52], [57], [61]. These different test environments make it difficult to compare the results. There is, however, one study in which the chamber size recommended for these aerosol experiments was used [75]. Our chamber, although smaller, has been previously used successfully for studying the aerosol stability of pathogens, including FCoV and bacteria (e.g., Escherichia coli) [45], [76].

### Limitations of the study

One limitation of this study is the inability to measure or control the exact radiation dose received by a virus particle while passing through the 275-nm UVC-LED chamber in our experimental setup. Investigating the impact of different radiation doses on virus reduction in aerosols would be valuable to identify the minimum effective dose required for successful inactivation. In addition, this study did not include a comparison of the energy consumption between the two technologies, which would be worthwhile in further studies in order to evaluate the energy efficiency of UVC-LEDs.

## Conclusion

Our study demonstrated the efficacy of a PAC equipped with a 275-nm UVC-LED unit or mechanical filtration for air decontamination against respiratory viruses in a defined setting. The combination of the two technologies showed the best performance. Our results suggest that emerging UVC-LED technology holds promise as a highly effective alternative for air decontamination. The required UVC dose for successful viral inactivation varies with wavelength, virus type, and environmental conditions, making the establishment of UV inactivation constants for bioaerosols complex and in need of further investigation. The deployment of PACs equipped with UVC radiation and filtration could be a promising alternative or supplement to ventilation systems, especially in healthcare settings and other public spaces where traditional ventilation systems may be inadequate for pathogen removal. Correct placement and operation of the device are crucial for optimal results. A standardized evaluation and testing of new air purification technologies should be pursued to ensure their effectiveness in mitigating airborne transmission of pathogens.

## Notes

### Authors’ ORCIDs

- Janina Reissner: 0009-0006-8467-7706
- Paul Siller: 0000-0002-0874-8473
- Gerrid Brockmann: 0000-0002-1600-7675
- Martin Kriegel: 0000-0003-0107-6333
- Uwe Roesler: 0000-0002-6268-5207
- Anika Friese: 0000-0002-2228-1101

### Funding

This work was financially supported by German Federal Ministry of Education and Research (grant number 03COV10G). 

### Acknowledgements

We would like to thank the German Federal Ministry of Education and Research for funding this work. Additionally, we would like to thank our project partners and colleagues at the Institute of Animal Hygiene and Environmental Health, Freie Universität Berlin, for their excellent technical support. 

### Competing interests

The authors declare that they have no competing interests.

## Figures and Tables

**Table 1:**
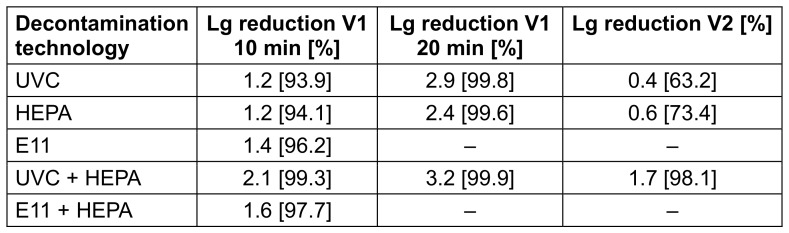
Lg reduction and reduction in % of the FCoV concentration by the different air decontamination technologies of the PAC and in different experimental setups in relation to the control

**Table 2:**
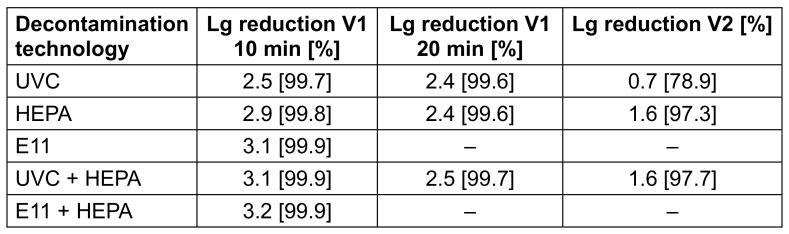
Lg reduction and reduction in % of the H3N2 concentration by the different air decontamination technologies of the PAC and in different experimental setups in relation to the control
